# Soil environment effects on the occurrence of black sesame spot and clubroot disease in Chinese Cabbage

**DOI:** 10.1371/journal.pone.0341603

**Published:** 2026-02-03

**Authors:** Xiuli Chi, Kun Yang, Chengran Li, Dong Chu

**Affiliations:** 1 Jiaozhou Agricultural and Rural Bureau, Qingdao, P. R. China; 2 Shandong Engineering Research Center for Environment-friendly Agricultural Pest Management, College of Plant Health and 24 Medicine, Qingdao Agricultural University, Qingdao, P. R. China; Universitat Jaume 1, SPAIN

## Abstract

Chinese cabbage is a popular leafy vegetable widely consumed in Asian cuisine, particularly in China. However, it is susceptible to various diseases, including clubroot and black sesame spot, which can significantly impact yield and quality. The soil environment plays a crucial role in developing these two diseases. In our experiment, we measured eight soil indicators-soil pH, organic matter, alkali-hydrolyzable nitrogen, available phosphorus, rapidly available potassium, total salinity, total nitrogen, and slowly available potassium-across 38 cabbage-growing locations in Jiaozhou County, China. We compared the soil environments of disease-infested and non-infested sites. Our results indicated that no significant association was found between the measured soil variables and observed clubroot incidence. However, there is a significant association between acidic soils with low available phosphorus and a higher incidence of black sesame spot. Overall, our study provides a comprehensive understanding of how the soil environment affects the occurrence of clubroot and black sesame spot, offering valuable insights for the integrated management of these challenging cabbage diseases.

## Introduction

Chinese cabbage, also known as Napa cabbage (*Brassica rapa* subsp. *pekinensis*), is a leafy vegetable that originates from East Asia and is commonly cultivated in this area. It is known for its elongated, light-green leaves forming dense, compact heads [1,2]. The plant grows best in cool climates, typically in early spring or fall, as high temperatures can cause the cabbage to bolt and produce flowers prematurely. It requires well-drained, fertile soil and adequate moisture to develop high-quality heads. Chinese cabbage is valued for its agricultural importance, as it is a significant crop in many countries [3,4].

Chinese cabbage has emerged as a significant agricultural product in Jiaozhou, China, due to the region’s favorable coastal climate. The moderate temperatures, humidity, and fertile soil create ideal growing conditions for this vegetable [5–7]. Chinese cabbage is susceptible to several diseases, including clubroot and black sesame spot, both of which can negatively affect yield and quality [8–10].

Clubroot disease, caused by the soil-borne pathogen *Plasmodiophora brassicae*, is one of the most destructive diseases affecting Chinese cabbage (*Brassica rapa* subsp. pekinensis) and other cruciferous crops. It is characterized by the formation of swollen, club-like galls on the roots, impairs the plant’s ability to take up water and nutrients [8]. This leads to stunted growth, wilting, yellowing of leaves, and, eventually, significant yield losses. The pathogen thrives in acidic soils (pH below 6.5), and its resting spores can persist in the soil for many years, which is difficult to manage [11–13].

Black sesame spot is a physiological disorder affecting Chinese cabbage’s white midrib tissues. The exact cause of this disorder remains unclear. While it is cosmetic and impacts the visual appeal and marketability of the cabbage, it is safe for consumption [14]. Black sesame spot symptoms are small, dark, circular or elongated spots on the outer leaves’ midribs and then spread to the middle inner leaves (Yang et al., 2006). The symptoms of black sesame spot would worsen after the Chinese cabbage’s storage for 10–12 days [15].

The soil environment is vital for many plant diseases, including clubroot and black sesame spot. The interactions between the soil environment and plant diseases are complex and influenced by biological, chemical, and physical factors. Soil health is critical for maintaining plant resilience against pathogens [8,16]. In this study, we compared the soil conditions at locations infested and uninfested with clubroot in Chinese cabbage and those infested and uninfested with black sesame spot based on the measurement of eight soil environment indicators. This study aims to elucidate the contrasting soil-environment relationships underlying a major pathogen-driven disease (clubroot) and a physiological disorder (black sesame spot), thereby providing targeted management insights.

## Materials and methods

### Chinese cabbage planted soil samples collected in different locations in Jiaozhou County

**Fig 1:**
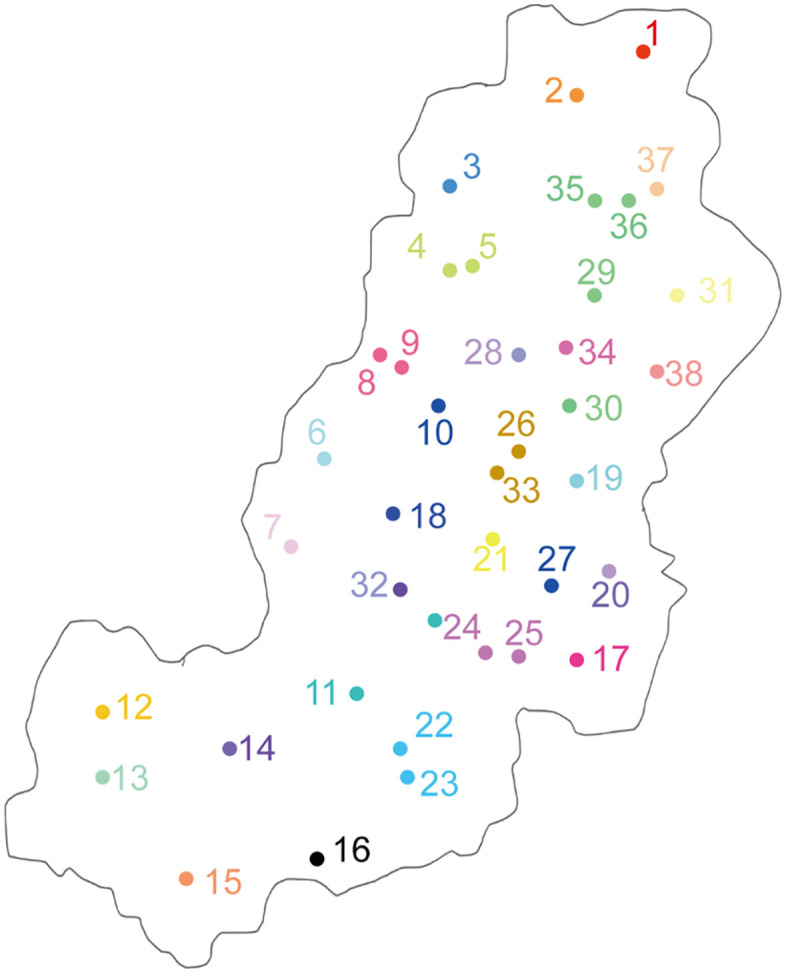
Collected areas of soil samples in different locations of Jiaozhou County, Shandong Province, China. Different numbers with different colors represent for different locations in Table 1.

A total of 38 soil samples from locations with Chinese cabbage planted in Jiaozhou County were collected (Fig 1). In most locations, only 1 soil sample was randomly collected except for the Right bank of Guhe Greenway, Yinjia Village, Beitai Village, Lengjia Village, Yangheya Village and Guanzhuang Village, in which areas 2 soil samples were collected. All soil samples were collected in December 2022, shortly after harvest, each location collected 500 g soil sample to monitor the soil environment indicators (Table 1). Before the soil sample monitoring, all soil samples were put in −20°C refrigerator. Clubroot disease identification was performed in a two-step protocol. First, field symptoms were assessed. Subsequently, for all samples from symptomatic plants, pathogen confirmation was carried out in the laboratory. Fresh galls were thoroughly washed, thin-sectioned, and stained with 0.1% trypan blue solution. The stained tissues were examined under a compound light microscope for the presence of abundant, spherical resting spore masses of *Plasmodiophora brassicae* within the root cortical cells. Only samples fulfilling both the macroscopic and microscopic criteria were recorded as positive for clubroot

**Table 1:** Soil environment of cabbage planted locations in Jiaozhou County in 2022.

Location number	Location in Jiaozhou	Soil pH	Organic matter (mg/kg)	Alkali-hydrolyzable nitrogen (mg/kg)	Available phosphorus (mg/kg)	Rapidly available potassium (mg/kg)	Total salinity (mg/kg)	Total nitrogen (mg/kg)	Slowly available potassium (mg/kg)
1	Pengjia Village	8.54	7800	24	23.1	139	700	380	427
2	South Jiaolai River Bridge	6.98	5080	35	16.2	184	800	370	632
3	Tanjiazhuang Village	6.04	10900	44	24.4	201	300	270	626
4	Right bank of Guhe Greenway1	7.17	6820	34	21.1	178	200	370	440
5	Right bank of Guhe Greenway2	7.44	6760	31	23.3	1.5	400	330	668
6	Xiaoxintuan Village	6.52	8420	36	19.6	175	400	350	552
7	Left Bank of Guhe Greenway	7.14	8750	28	19.1	172	400	380	269
8	Yinjia Village1	6.01	10400	101	25.4	154	NA	990	522
9	Yinjia Village2	5.8	13000	91	24.8	169	NA	1170	614
10	Luojia Village	6.8	25900	101	14.1	201	800	1510	443
11	Qianshilong Village	7.86	23800	77	40.2	558	2200	1550	705
12	Jiatuan Village	4.47	27400	172	22.7	231	2600	1200	538
13	Qianxintuan Village	5.7	19200	161	23.6	193	2400	1120	356
14	Qianshilong Village	6.46	17400	77	20.7	167	1700	1220	627
15	Qianfenglongtun Village	6.3	23100	311	24.4	195	2000	1710	347
16	Xiegouya Village	6.34	27800	188	25.7	183	2400	1730	334
17	Xiaogaojia Village	6.01	15500	103	22.5	173	2700	1010	463
18	Nanwangtuan Village	6.46	12200	117	23.4	258	2300	830	587
19	Wangjiahe Village	6.3	15000	75	22.2	247	2200	1050	340
20	Nandianzi Village	7.21	17900	86	32.2	138	1300	1260	382
21	Taijia Village	5.63	14400	101	23.2	169	2700	1240	219
22	Beitai Village1	6.92	21600	130	37.9	271	1200	1320	415
23	Beitai Village2	7.41	16100	110	35.4	270	2300	1150	261
24	Lengjia Village1	6.02	11000	51	22.7	119	1800	870	539
25	Lengjia Village2	6.01	9500	73	22.3	110	2800	830	483
26	Yangheya Village1	6.01	17000	164	24.7	485	1700	1200	552
27	Xuejiahe Village	5.98	17500	123	27.3	189	2200	1220	594
28	Dongbu Village	7.1	16000	141	27.1	106	1700	1250	699
29	Xiaofole Village	5.67	12900	494	23.6	326	2400	1430	433
30	Yuangezhuang Village	6.26	8900	71	21.8	107	2400	820	429
31	Zhangjiazhuang Village	5.38	15100	67	25.3	125	2000	900	536
32	Pengjiazhuang Village	5.42	11900	54	26.6	220	1600	930	291
33	Yangheya Village2	5.32	12000	51	25.7	109	1700	910	558
34	Shiwuli Village	6.73	8200	286	39.4	68	2100	700	69
35	Guanzhuang Village1	6.36	18000	63	21.8	111	2400	910	284
36	Guanzhuang Village2	6.36	31300	145	19.8	521	2100	2010	632
37	Balizhuang Village	5.73	26100	172	20.3	177	2500	1300	427
38	Lijiatun Village	7.06	17300	71	32.1	187	2800	1220	657

Note: NA indicates data not available. Clubroot disease infestation in 2022: locations 1–31 were not infested; locations 32–38 were infested. Black sesame spot disease infestation in 2022: locations 2, 18, 21, 27, 30, 32, 35–37 were infested; locations 11, 14, 16, 20, 26, 38 were not infested.

### Soil environmental monitoring of different soil samples in Jiaozhou

Eight key soil chemical properties were determined: soil pH, organic matter, alkali-hydrolyzale nitrogen, phosphorus, rapidly available potassium, total salinity, total nitrogen, and slowly available potassium.

### Soil pH measurement

Soil pH was measured by glass electrode following the protocols of Fornasier et al. [17], using a pH Meter, Glass Electrode, Saturated Calomel Electrode or pH Composite Electrode, and Stirrer. The electrode was immersed in the soil suspension, and the beaker was gently rotated. The pH value was recorded after stabilization. Between measurements, the electrode was rinsed with water and dried with filter paper. Calibration was verified with a standard solution after every 5–6 samples.

### Soil organic matter

Under heating conditions, an excess of potassium dichromate-sulfuric acid solution oxidizes soil organic carbon. The excess potassium dichromate is titrated with a standard solution of ferrous sulfate. The amount of consumed potassium dichromate is calculated to determine the organic carbon content using the oxidation correction factor, which is then multiplied by the constant 1.724 to yield the soil organic matter content.

### Soil alkali-hydrolyzale nitrogen measurement

Treating soil with an alkaline solution, easily hydrolyzed organic nitrogen and ammonium nitrogen are converted to ammonia. Nitrate nitrogen is first reduced to ammonium nitrogen and then to ammonia. The ammonia diffuses and is absorbed by boric acid, which is then titrated with standard acid to calculate the content of alkaline-hydrolyzed nitrogen.

### Soil available phosphorus measurement

Using a constant temperature chamber, reciprocating shaker, spectrophotometer, plastic bottle (150 mL), and colourimetric tube (25 mL). Sodium bicarbonate extractant extracts available phosphorus from neutral and calcareous soils, while ammonium fluoride and hydrochloric acid extractant are used for extracting available phosphorus from acidic soils. The air segment continuous flow analysis technique is employed to uniformly mix the sample solution and reagents in a continuous flow system, develop color, and determine the mass concentration of available phosphorus in the sample solution at a specific wavelength, thereby calculating the available phosphorus content in the soil samples.

### Soil rapidly available potassium

With a reciprocating shaker (with an oscillation frequency of 150 r/min to 180 r/min) and flame photometer, soil-available potassium is extracted using a neutral 1 mol/L ammonium acetate solution and measured with a flame photometer.

### Soil total salinity

Total soluble salt content was measured by the weight method of the dry residue. Briefly, salts were extracted from soil with water (1:5, w/v) via shaking. The extract was filtered, evaporated, and the dried residue was treated with hydrogen peroxide to oxidize organic matter. The weight of the remaining residue represented the total soluble salt content.

### Soil total nitrogen

Total nitrogen was measured following the Kjeldahl digestion method. Briefly, soil samples were digested at high temperature (>400°C) with concentrated H₂SO₄ and a catalyst to convert all nitrogen to ammonium. The ammonium was then distilled, absorbed in boric acid, and titrated automatically to determine the content.

### Soil slowly available potassium

Using a flame photometer, oil bath, or phosphoric acid bath, with soil extracted using 1 mol/L hot nitric acid, determines the acid-soluble potassium content via the flame photometer. The content of slow-release potassium is obtained by subtracting the readily available potassium content.

### Data analysis

The experimental unit for all analyses was an individual field location (n = 38). Each soil measurement was conducted in triplicate (technical replicates), and the mean value was used for statistical analysis to ensure measurement precision. The Shapiro–Wilk test (SPSS 21.0) was performed to measure whether the soil environment indicator data followed a normal distribution (Table 1). As the soil environment indicator data all followed a normal distribution, Student’s t-test (SPSS 21.0) was used to analyze the significant differences of soil environment indicator data between disease-infested and un-infested soil samples. Principal Component Analysis of occurrence and no occurrence of the two diseases were measured by R languages (version 4.4.0).

## Results

### Soil environment varied in different locations in Jiaozhou County with different cabbage diseases

The soil environment of 38 Chinese cabbage planted locations in Jiaozhou was examined after the cabbage was harvested, including the soil pH, organic matter, alkali-hydrolyzale nitrogen, available phosphorus, rapidly available potassium, total salinity, total nitrogen, and slowly available potassium. In all locations, soil pH ranged from 4.47 to 8.54. Organic matter ranged from 5080 to 313000 mg/kg, alkali-hydrolyzale nitrogen ranged from 24 mg/kg to 494 mg/kg, available phosphorus ranged from 14.1 mg/kg to 40.2 mg/kg, rapidly available potassium ranged from 1.5 mg/kg to 558 mg/kg, total salinity 200 mg/kg to 2800 mg/kg, total nitrogen ranged from 270 mg/kg to 2010 mg/kg, slowly available potassium ranged from 69 mg/kg to 705 mg/kg, respectively (Table 1).

In all soil-collected locations, Chinese cabbages planted in locations 1–31 were not infested with clubroot disease. However, cabbages planted in locations 32–37 were infested with clubroot in 2022. Additionally, cabbages in locations 2, 18, 21, 27, 30, and 32, as well as locations 35–37, were infested with black sesame spot disease. In contrast, cabbages planted in locations 11, 14, 16, 20, 26, and 38 were not infested with black sesame spot disease in 2022 (Table 1). Disease incidence was assessed by visual inspection and microscopic examination. This broad variation in soil baseline conditions across the surveyed region provided a suitable foundation for investigating potential associations with disease incidence.

### Relationship between the occurrence of cabbage black sesame spot and soil environment in the Jiaozhou area

**Fig 2:**
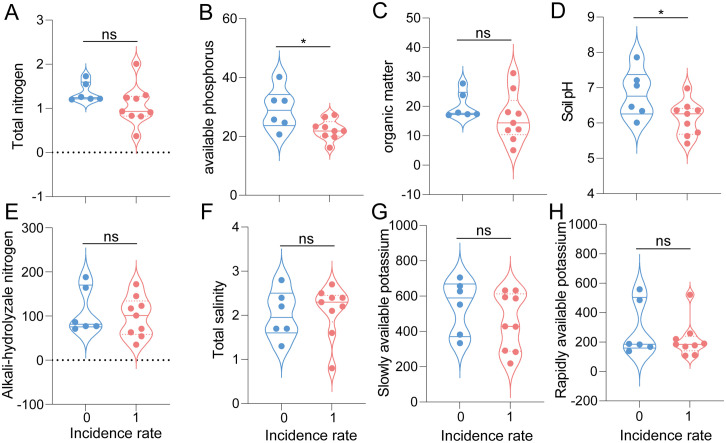
Soil environment indicators of black sesame spot diseases incidence and un-incidence area in Jiaozhou County, including total nitrogen (A), available phosphorus (B), organic matter (C), soil pH (D), alkali-hydrolyzale nitrogen (E), total salinity (F), slowly available potassium (G) and rapidly available potassium (H), respectively. ns: not significant; *: P < 0.05.

**Fig 3:**
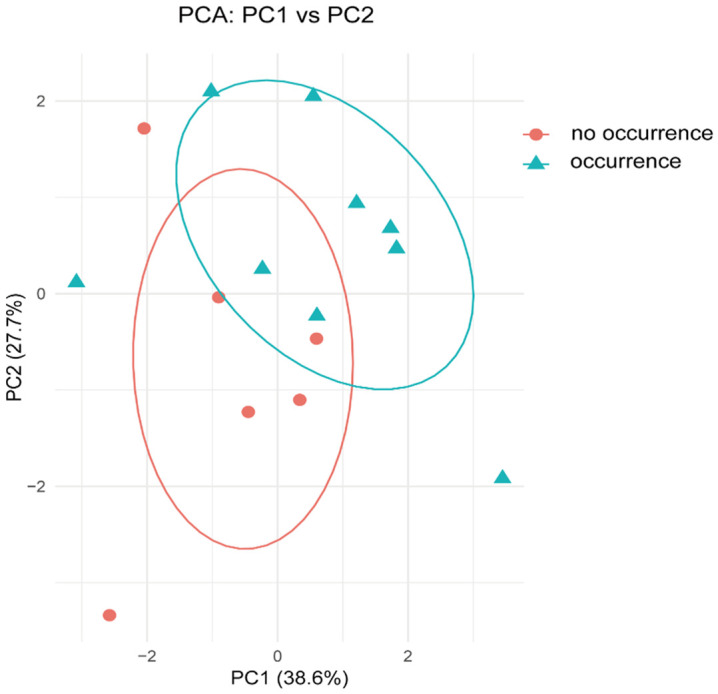
Principal Component Analysis of occurrence and no occurrence of black sesame spot diseases in Jiaozhou County with different environmental factors.

The soil environment indicators were compared between black sesame spot diseases infested and uninfected locations in Jiaozhou County (Table 1). Based on the results, total nitrogen, organic matter, alkali-hydrolyzale nitrogen, total salinity, slowly available potassium and rapidly available potassium data were not significantly different between soil samples of black sesame spot diseases infested and un-infected locations (*P* > 0.05, Student’s t-test) (Fig 2), while for available phosphorus data, black sesame spot diseases infested soil (22.27 ± 1.14, Mean ± SEM) was significantly lower than that of un-infested soil (29.27 ± 2.85, Mean ± SEM) (t_0.025/13_ = −2.61, *P* < 0.05, Student’s t-test) (Fig 2B), and data of soil pH of soil samples of black sesame spot diseases infested locations (6.13 ± 0.16, Mean ± SEM) was significantly lower than that of un-infested locations (6.82 ± 0.28, Mean ± SEM) (t_0.025/13_ = −2.31, *P* < 0.05, Student’s t-test) (Fig 2D). Based on Principal Component Analysis (PCA), these results indicate a clear statistical association between the incidence of black sesame spot and two specific soil factors: lower pH and reduced available phosphorus (Fig 3).

### Relationship between the occurrence of cabbage clubroot disease and soil environment in Jiaozhou area

**Fig 4:**
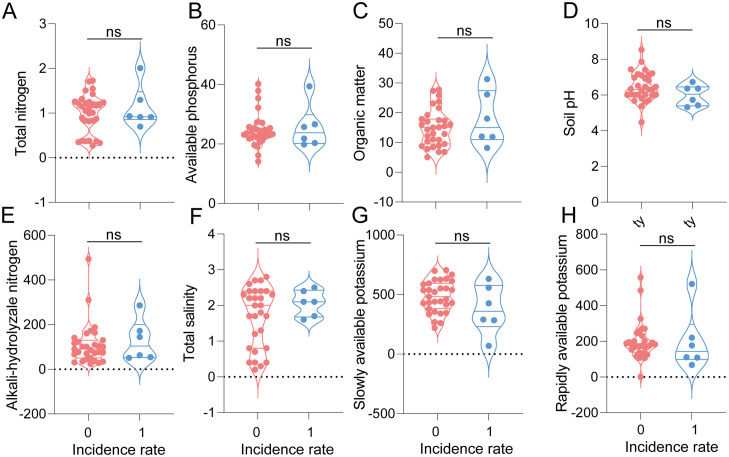
Soil environment indicators of clubroot diseases incidence and un-incidence area in Jiaozhou County, including total nitrogen (A), available phosphorus (B), organic matter (C), soil pH (D), alkali-hydrolyzale nitrogen (E), total salinity (F), slowly available potassium (G) and rapidly available potassium (H), respectively. ns: not significant.

**Fig 5:**
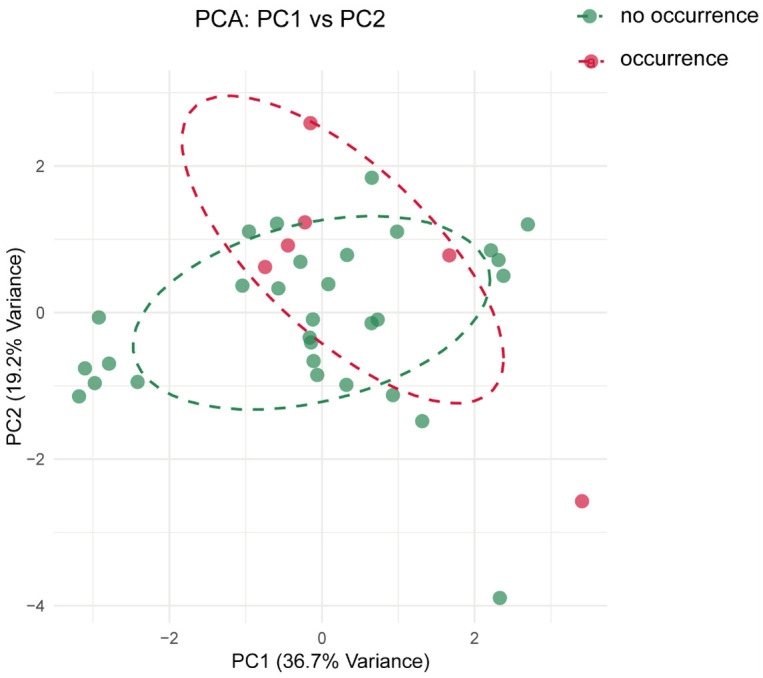
Principal Component Analysis of occurrence and no occurrence of clubroot diseases in Jiaozhou County with different environmental factors.

The soil environment indicators were compared between clubroot diseases infested and uninfected locations in Jiaozhou County (Table 1). Based on the results, all the soil environment indicators, including soil pH, organic matter, alkali-hydrolyzale nitrogen, available phosphorus, rapidly available potassium, total salinity, total nitrogen, and slowly available potassium, were not significantly different between soil samples of clubroot diseases infested and uninfected locations (*P* > 0.05, Student’s t-test) (Fig 4). Contrary to the well-established relationship between clubroot and soil acidity, our analysis did not detect a significant association between the measured soil chemical properties and disease incidence in this field survey (Fig 5).

## Discussion

In this study, we analyzed eight soil indicators-soil pH, organic matter, alkali-hydrolyzable nitrogen, available phosphorus, rapidly available potassium, total salinity, total nitrogen, and slowly available potassium-across 38 cabbage-growing locations in Jiaozhou County, China. We compared the soil environments of disease-infested and non-infested sites and found that soil conditions did not influence the occurrence of clubroot disease. However, our survey identifies a significant association between acidic soils with low available phosphorus and a higher incidence of black sesame spot. This correlation does not imply causation; rather, these soil conditions may represent key risk factors or components of a conducive environment for the expression of this complex physiological disorder, the precise etiology of which remains unclear. Supporting these findings, another study reported that soil available phosphorus increased from 17.09 mg/L in the 1990s to 33.28 mg/L in the 2000s [18], consistent with our results, which showed available phosphorus levels ranging from 14.1 mg/kg to 40.2 mg/kg (Table 1), further validating the credibility of our findings.

Soil plays a crucial role in plant health and development, serving as a nutrient reservoir and a medium for growth. However, it can also harbour pathogens, creating an environment conducive to various plant diseases. The interaction between soil conditions and plant pathogens is complex. Factors such as moisture content, pH, temperature, and organic matter influence the survival and virulence of disease-causing organisms [19,20]. Among the many soil-borne diseases, clubroot and black sesame spot are prominent examples that significantly impact crop health and yield [8,12]. Exploring the soil environment before planting cabbage is important for disease control.

Although black sesame spot is a physiological disease with no exact cause identified [14], the soil environment, particularly nitrogen levels, is crucial for the occurrence of this disease. Ammonium nitrogen had a more significant influence on the occurrence of black sesame spots compared to nitrate nitrogen. Different NO_3_^-^-N:NH_4_^+^-N ratios also affected the prevalence of black sesame spots [21,22]. Another study showed a close relationship between the occurrence of black sesame spot and cupric ions [23]. In the current study, no relationship was detected between black sesame spot morbidity and soil nitrogen content (Fig 2A). However, there was a significant correlation between black sesame spot occurrence and available phosphorus (Fig 2B) as well as soil pH (Fig 2D).

As a devastating soil-borne disease caused by the pathogen *Plasmodiophora brassicae*, clubroot morbidity is heavily influenced by soil conditions. For example, moist and acidic soils (pH below 7) provide an optimal environment for *Plasmodiophora brassicae* to thrive, while drier soils tend to limit the pathogen’s ability to spread [24,25]. Other factors, such as soil temperature, organic matter content, and calcium or magnesium, contribute to the pathogen’s survival and disease expression [8]. However, no significant association was detected between the measured soil indicators and clubroot incidence in this study (Fig 3), a finding that may be attributed to the constraints of our limited sample size (n = 38 locations in Jiaozhou). Soil phosphorus is an essential nutrient that plays a significant role in plant health and development. Its availability in the soil can influence the occurrence and severity of various plant diseases [26]. For example, adequate phosphorus levels promote healthy root development, enhancing the plant’s overall vigour and disease resistance [27]. Some pathogens can be more aggressive in low-phosphorus conditions, such as plants deficient in phosphorus may exhibit stunted growth and weakened defence, making them more susceptible to infections from pathogens like Phytophthora and Fusarium species [28,29]. In this study, low phosphorus levels may reduce cabbage resistance to black sesame spot disease (Fig 2B).

Soil pH plays a critical role in the development and severity of various plant diseases. It can influence pathogen survival, virulence, and plant susceptibility to infections [30]. For instance, essential nutrients such as nitrogen, phosphorus, and potassium are more readily available in neutral to slightly alkaline soils. Additionally, soil pH influences the composition and activity of the microbial community in the soil [20,31]. We therefore hypothesize that soil acidity creates a predisposing environment for the disorder, possibly through these integrated pathways.

An important methodological consideration in this study is the sampling design. With predominantly one soil sample collected per location, our data may not comprehensively capture the within-field spatial variability of soil parameters. This sampling intensity, while providing a broad survey across the region, could potentially mask localized hotspots of conducive or suppressive conditions for disease development. Consequently, our analysis reflects field-average conditions, and the strength of association between soil factors and disease incidence might be attenuated. Future studies aiming to establish precise, site-specific management recommendations would benefit from a more intensive sampling scheme, such as collecting multiple, georeferenced subsamples per field to create a composite sample.

Taken together, we measured eight soil indicators across 38 cabbage-growing locations in Jiaozhou County, China, comparing disease-infested and non-infested sites. Our results showed that no significant association was found between the measured soil variables and observed clubroot incidence, whereas black sesame spot was more prevalent in acidic soils with low phosphorus levels. This study offers valuable insights into the effects of soil environment on these cabbage diseases, aiding in their integrated management.
